# Assessment of dietary patterns, physical activity and obesity from a national survey: Rural-urban health disparities in older adults

**DOI:** 10.1371/journal.pone.0208268

**Published:** 2018-12-05

**Authors:** Steven A. Cohen, Mary L. Greaney, Natalie J. Sabik

**Affiliations:** Health Studies Program, Department of Kinesiology, University of Rhode Island, Kingston, Rhode Island, United States of America; Universidad del Desarrollo, CHILE

## Abstract

**Background:**

Obesity is a critical public health issue, affecting over one-third of all Americans, and is an underlying cause of numerous health issues across the lifespan. For older adults, obesity is linked to premature declines in physical and mental health and cognitive functioning. The occurrence of obesity and related health behaviors and chronic diseases are higher in rural areas than in urban areas. Furthermore, rural areas of the United States have a higher proportion of older adults than urban areas. Few studies, to date, have explored rural-urban differences in the relationships between dietary patterns and obesity among older adults. Therefore, the purpose of this study is to assess rural-urban differences in obesity rates in older adults, and the potential for the associations between obesity and physical activity and dietary patterns to vary by rural-urban status.

**Methods:**

Data were abstracted from respondents aged 65 and above from the 2012 Behavioral Risk Factor Surveillance System (BRFSS) database linked to Census-based county-level information on rural-urban status and socioeconomic status. Generalized linear models were utilized to assess rural-urban disparities in obesity, and the potential for associations between obesity and known risk factors (fruit consumption, green vegetable consumption and physical activity) to vary by rural-urban status, accounting for complex sampling and confounders.

**Results:**

Obesity rates were highest and fruit consumption was lowest in the most rural areas. However, for older adults in the most urban areas, there was a significant negative association between obesity and fruit and green vegetable consumption. This association was not observed in more rural older adults.

**Conclusion:**

These findings underscore the need to take into account place-based factors such as rural-urban status, when designing and implementing policies and interventions designed to reduce obesity through risk factor mitigation in older adults. To reduce rural-urban disparities in older adults, all policies, programs, and interventions should address the unique barriers and needs specific to rural and urban older adults.

## Background

More than one third of the United States (U.S.) population aged 65 and above (35%) are affected by obesity [1], and nearly one in 7 older adults have a body mass index (BMI) of 35 kg/m^2^ or higher. From 2003 to 2012, the prevalence of obesity (BMI ≥ 30) in older adults increased by 4.4 percentage points, surpassing the growth rate of obesity in all other age groups [2]. Across the lifespan, obesity reduces quality of life and causes substantial health complications, such as type 2 diabetes and hypertension [3,4]. Obesity, however, is particularly problematic in older adults, as it promotes premature frailty and exacerbates age-associated declines in physical and cognitive functioning and mental health [4]. Furthermore, obesity significantly increases all-cause mortality among older adults [5–7]. The problem of obesity in older adults will increase in severity and scope as the population age 65 and above in the U.S. will increase from 47.8 million in 2015 to 82.3 million by 2040, an increase from 13% to 20% of the total U.S. population [8].

The central causes of obesity across the age spectrum [9–14] and in older adults [5,15] are well-documented, and include unhealthful dietary habits, lack of physical activity, and other behavioral and environmental factors, such as the particular aspects of the built environment and neighborhood characteristics. Over the past several decades, obesity research has explored numerous correlates and causes of obesity, including socioeconomic, demographic, genetic, biological, medical, and institutional factors [16–20]. With respect to socioeconomic and demographic factors, a study of older adults indicated that obesity prevalence is highest among Blacks, females, and individuals with lower educational attainment [21].

Research, however, suggests that obesity risk depends not only on individual factors, but also on place-based, area-level factors, such as community infrastructure, socioeconomic conditions, demographics, environmental, and other community-specific factors which exacerbates the challenge of obesity prevention [22–24]. Above and beyond the contributions of individual factors, community-level, place-based factors such as poverty and an environment that promote overeating and sedentary behaviors may play a substantial role in obesity [25] and serve as one of the primary causes of many health disparities across the lifespan [26]. For example, previous studies have found a strong and consistent association between low community socioeconomic status and increased density of fast food outlets [21,27,28], and reduced availability of healthier food options, such as fresh fruits and vegetables in so-called “food deserts” [29,30]. Similarly, areas with high poverty rates have been found to be associated with reduced levels of perceived safety and walkability and resultant physical inactivity and other sedentary behaviors [31,32]. Rural-urban differences in obesity are also apparent. Among adults aged 20–39, the prevalence of obesity was significantly higher in rural areas (38.1%) than in urban areas (27.9%), although the corresponding difference in older adults was not significantly [33].

Nonetheless, place-based factors contributing to obesity have been underexplored in older adults, despite the fact that this information is an essential to understanding disparities in obesity and associated health issues in older adults. Despite the magnitude of the obesity epidemic and its substantial effects on health, the scientific and health care communities have struggled to find and implement effective, population-based approaches for promoting healthy weight and preventing comorbidities in older adults [16].

This study explores a potentially important place-based factor—rural-urban status—known to contribute to health disparities across the lifespan, including in older adults [34]. For example, rural residents are more likely to have obesity [35, 36] and related chronic diseases [37] compared to their urban counterparts [33]. A recent study of women aged 40 and older found that women living in rural areas, especially those living in the rural South, and who had less education, were more sedentary, and reported more personal barriers to physical activity than women in urban areas [38, 39]. Since rural areas of the U.S. generally contain a higher proportion of older adults than urban areas [40], obesity among older adults may be more of a pressing concern in rural areas. Aging in rural areas carries unique challenges such as seeking transportation to medical and dental appointments, grocery shopping, and other essential activities for successful aging, such as leisure, enrichment, places to exercise, and the built environment (e.g. lack of sidewalks, etc.) [41]. Furthermore, older adults in rural areas have difficulty securing needed home and community-based services and long-term care in their communities [42].

Yet, no study to date has systematically examined differences in the prevalence of obesity and factors associated with obesity in older adults by rural-urban status. Furthermore, few studies have addressed the potential for the associations between well-established causes of obesity and obesity to vary based on rural-urban status in older adults. Therefore, the primary objective of this study was to assess the association between rural-urban status and physical activity, fruit and green vegetable consumption, and obesity in older adults. The secondary study objective was to explore how those potential associations vary by rural-urban status.

## Methods

### Data sources and sample

This is a secondary analysis of data from the 2012 Behavioral Risk Factor Surveillance System (BRFSS), the largest system of health-related telephone surveys administered by the Centers for Disease Control and Prevention (CDC). BRFSS collects data from U.S. residents in all 50 states regarding their demographics, self-reported health-related risk behaviors, height, weight, chronic health conditions, and use of preventive services annually, and is used for planning and prevention efforts [43]. Over 400,000 interviews with BRFSS respondents age 18 and above are conducted each year. The 2012 BRFSS sample was selected for this study as it was the most recent year in which respondent’s county of residence is available in the public-use dataset.

The 2012 BRFSS included 475,687 total respondents, with response rates for landline and cell phone based being 49.1% and 35.3% [44], respectively, of which 152,541 (32.1%) were aged 65 and over and are included in the analytic sample for this study. The analytic sample for the current study was restricted to those aged 65+ who provided information on height, weight, and all other key study variables living in the contiguous U.S. Each of these respondents was linked to area-level data from the 2010 U.S. Census via county Federal Information Processing Standard (FIPS) code. All data were de-identified prior to public release, so confidentiality could be maintained throughout the analysis.

### Measures

#### Obesity

Height and weight was used to calculate BMI and used to determine weight status. Respondents whose BMI was 30 kg/m^2^ or above were classified as having obesity.

#### Physical activity

Physical activity was assessed by ne question: “During the past month, other than your regular job, did you participate in any physical activities or exercises such as running, calisthenics, golf, gardening, or walking for exercise?” Respondents responded either “yes”, “no”, “don’t know/ not sure”, or refused to answer. Those who answered “don’t know/ not sure” or who refused to answer were coded as missing, and the remainder were coded as a binary measure of participation in physical activity in the past month. Information on physical activity participation was available for 151,956 (99.6%) of the respondents in the analytic sample.

#### Fruit and green vegetable consumption

Fruit and green vegetable consumption was asked in an optional state-based module in seven states (Arizona, California, Delaware, Georgia, Maryland, Ohio, and Tennessee). To estimate fruit consumption, respondents were asked: “During the past month, not counting juice, how many times per day, week, or month did you eat fruit? Count fresh, frozen, or canned fruit.” Similarly, for green vegetable consumption, respondents were asked: “During the past month, how many times per day, week, or month did you eat dark green vegetables for example broccoli or dark leafy greens including romaine, chard, collard greens or spinach?” For each question, respondents estimated eating occasions per day, week, or month. All responses were then converted into a continuous measure of fruit (or green vegetable) eating occasions per week, and used to assess the associations between rural-urban status and physical activity, fruit and green vegetable intake, and obesity in older adults. To explore how the associations between obesity and fruit intake, green vegetable intake, and participation in physical activity potentially vary by rural-urban status, the fruit and green vegetable consumption variables were dichotomized into “high” or “low” consumption based on their respective median values to facilitate interpretation of results.

#### Rural-urban status

Population density is a widely-used measure of rural-urban status in the public health literature [35]. County-level population density from the 2010 US Census was linked to each BRFSS respondent’s county of residence and used as the central measure of rural-urban status and binned into quintiles based on all US counties. Respondents were more likely to live in more urban counties than rural counties, so the distribution of BRFSS respondents by population density quintile was uneven. For instance, approximately 57% (76,847) of the respondents for which population density was available (134,536) were in the most urban quintile of population density. However, the measure used was stratified into quartiles internals across the BRFSS analytic sample, meaning that there were approximately equal numbers of respondents in each rural-urban stratum (33,512–33,802 per stratum).

#### Covariates and confounders

Confounders and covariates examined in this study included race (white versus non-white), gender, education level (high school diploma or higher versus less than high school education), personal income, age, and county-level per capita income.

### Data analysis

Descriptive statistics were obtained for all variables of interest, including frequencies for categorical variables and means and standard deviations or medians and interquartile ranges for continuous variables. Chi squared tests were used to assess bivariate associations between categorical variables, and t-tests, ANOVA, Wilcoxon Rank Sum and Friedman tests assessed bivariate associations between pairs of categorical and continuous variables. Geographic information systems (GIS) was used to descriptively map the distributions of several key variables and sample characteristics used in the analysis.

To complete the first research objective—to assess how obesity, physical activity, fruit intake, and green vegetable consumption are related to rural-urban status in older adults—each of these four variables was modeled against rural-urban status quintile using generalized linear models with a logistic link function to account for confounders and incorporate complex sampling. The most urban quintile of population density (Q5) was used as the reference group. A trend test also was conducted for each of the four outcomes to assess the potential for a monotonic relationship, one in which the relationship between the measures varies in such a way that it either never decreases or never increases, between each of the outcomes and rural-urban status in older adults.

To address the second research objective—to assess whether the associations between predictors of obesity and obesity vary by rural-urban status—generalized linear models were used to model the outcome of obesity separately on each of the three predictor variables (fruit consumption, green vegetable consumption, and physical activity), accounting for potential confounders and complex sampling. The models were conducted with the entire analytic sample for which each of the four main variables of interest were available, and stratified by population density quintile to explore potential differences by rural-urban status. SAS version 9.4 (Cary, NC) and IBM SPSS version 24 (Armonk, NY) were used for data management and analysis, and ArcGIS version 10 (Redlands, CA) was used for all mapping.

## Results

**Fig 1:**
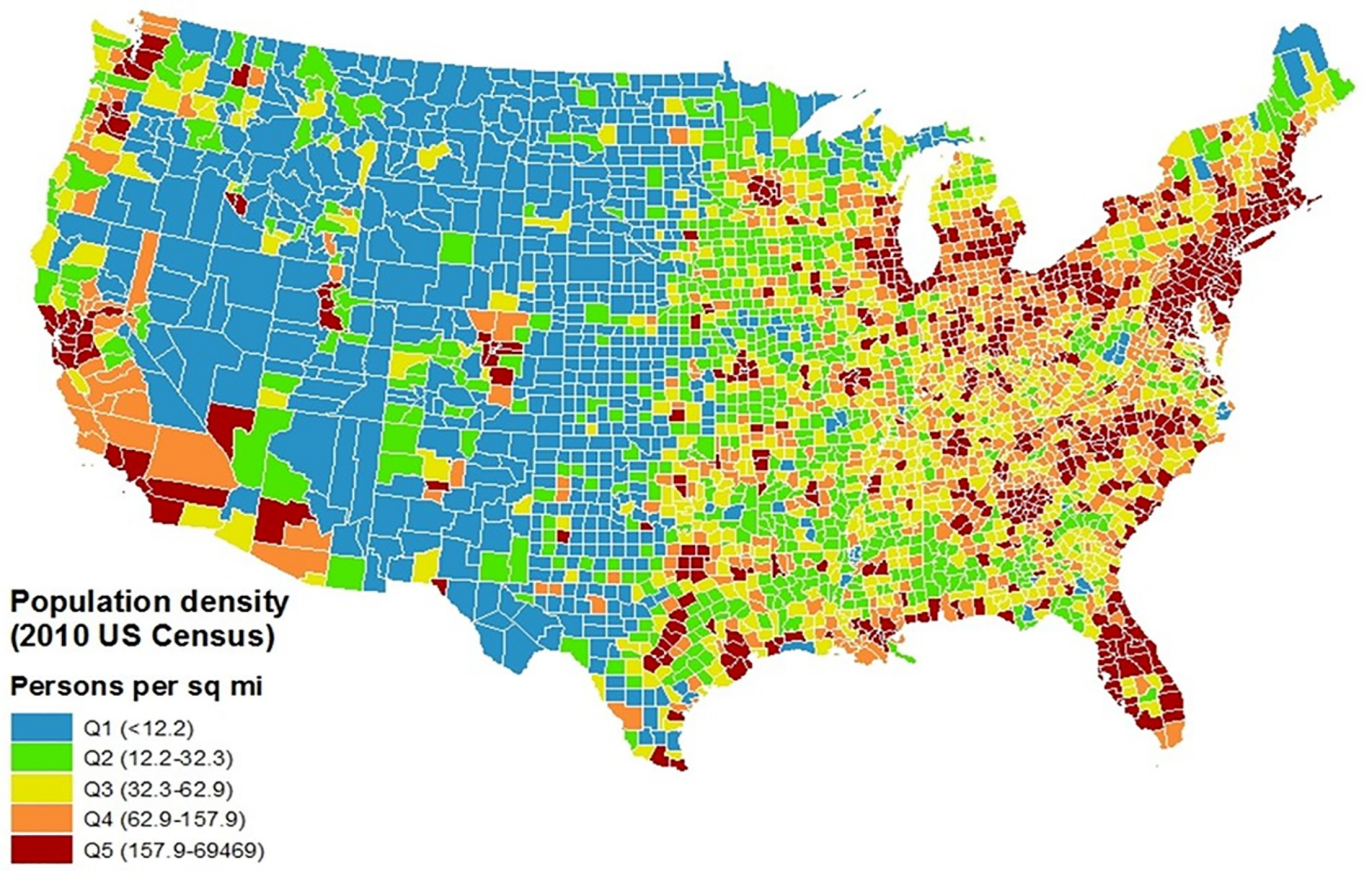
Geographic distribution of population density quintile by county.

**Fig 2:**
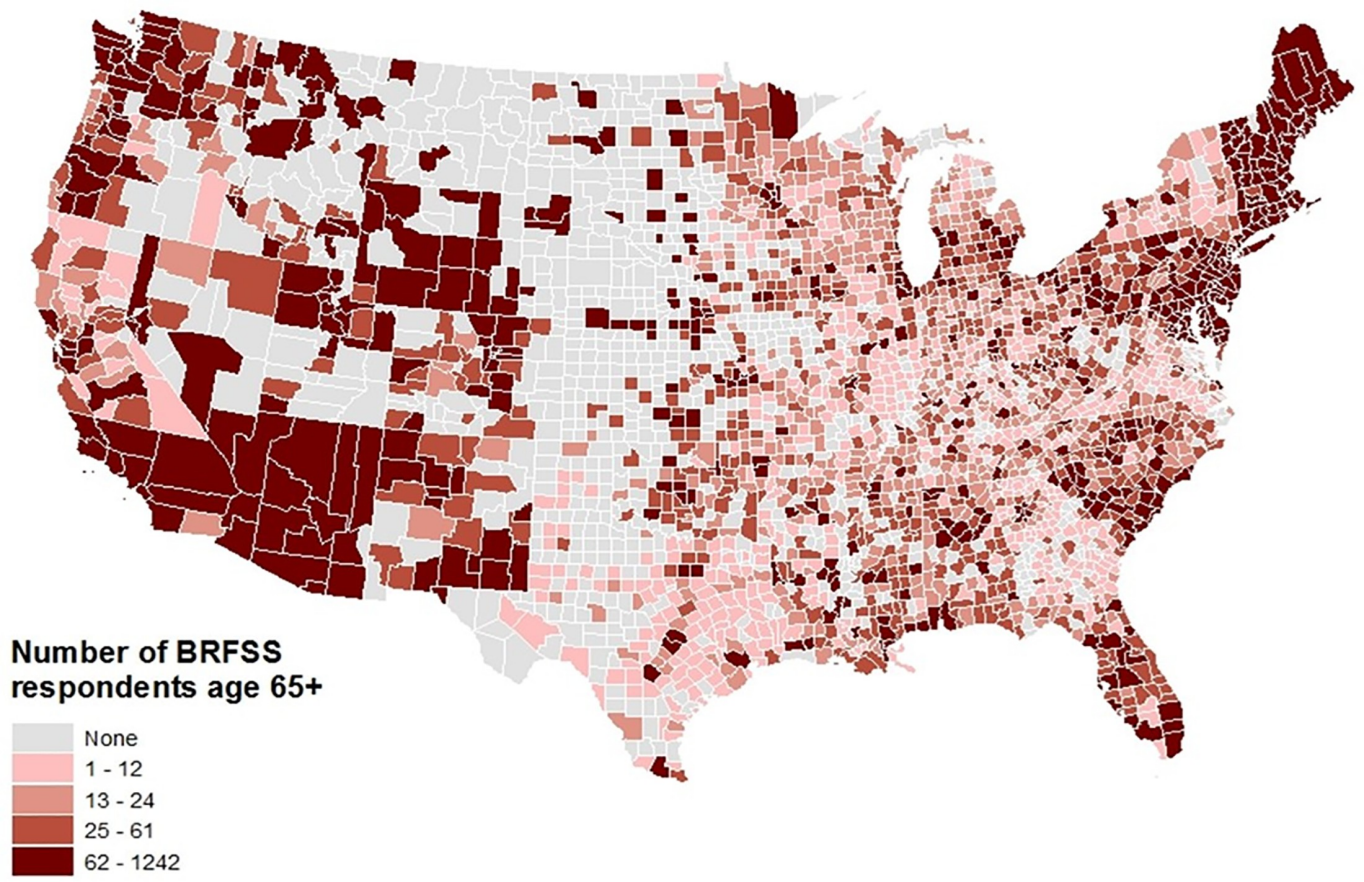
Geographic distribution of the analytic sample: Number of respondents aged 65 and above from the 2012 Behavioral Risk Factor Surveillance System.

**Fig 3:**
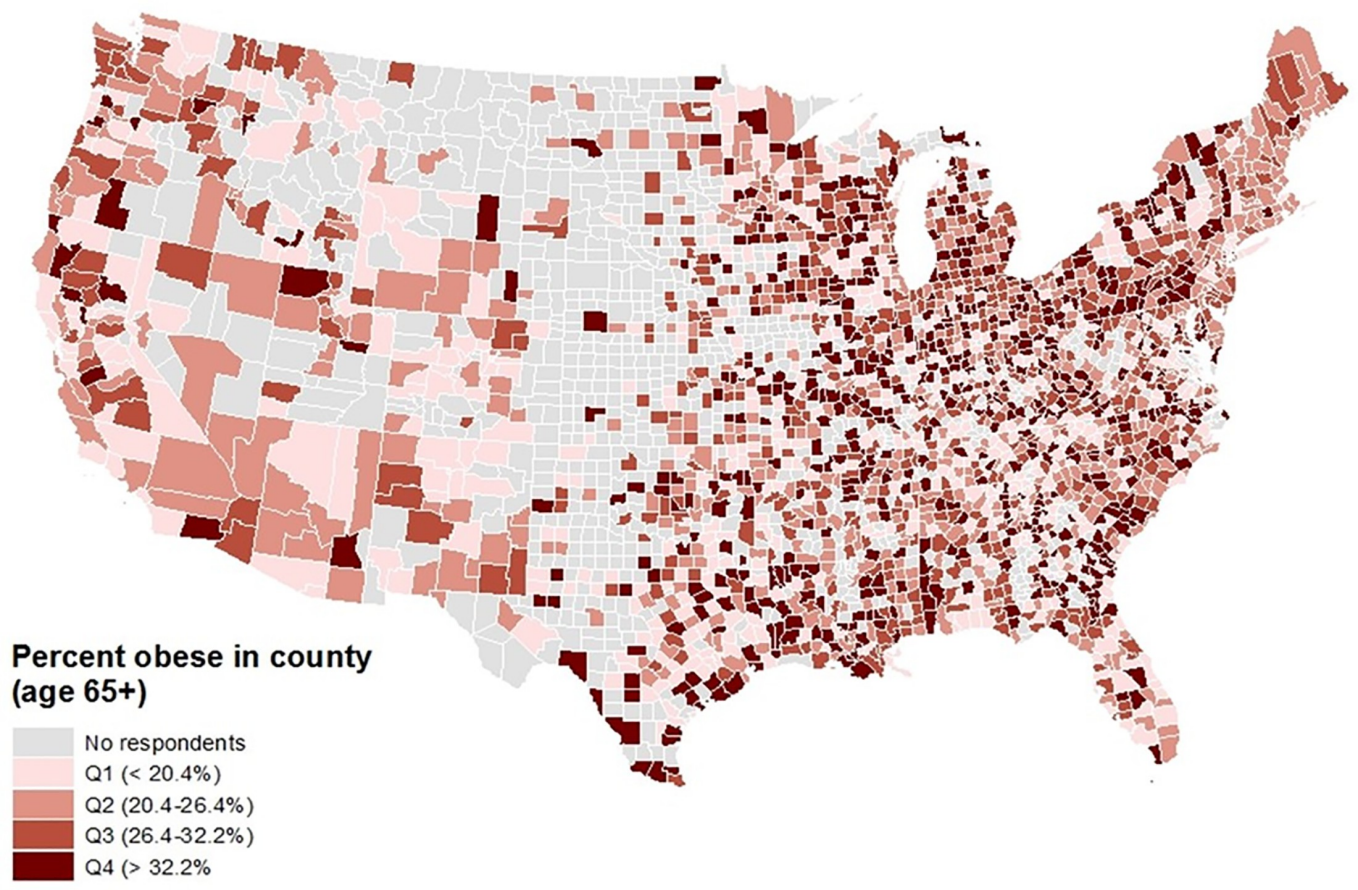
Geographic distribution of obesity prevalence in the analytic sample: Percent of respondents age 65+ who are obese (BMI ≥ 30, by quartile of obesity prevalence).

As shown in Table 1, the majority of the respondents were White (88.0%) and female (62.6%), had at least a high school education (87.9%), and had annual household incomes less than $50,000 (76.6%). Respondents’ average age was 74.2 years, with a standard deviation of 6.6 years, and most lived in more urban than rural areas. Respondents with obesity were significantly more likely to be non-white, to have less education, be older, have an income less than $50,000 per year, live in a poorer county, and were less likely to have been physically active and to eat fruits and green vegetables. Fig 1 shows the distribution of population density quantile by county, Fig 2 depicts the number of respondents in the analytic sample by county, and Fig 3 shows the percent of respondents classified as having obesity by county.

**Table 1:** Descriptive statistics for the analytic sample by obesity status.

		Weight Status		
		Obese	Non-obese	P-value
		*N (%)*	*N (%)*	
Race	White	31321 (24.3)	97325 (75.7)	< 0.001
	Non-white	5610 (32.0)	11948 (68.0)	
Sex	Female	23093 (25.2)	68406 (74.8)	0.825
	Male	13847 (25.3)	40905 (74.7)	
Education	HS or greater	31433 (24.4)	97245 (75.6)	< 0.001
	LT HS	5507 (31.3)	12066 (68.7)	
Income	≥ $50,000	7725 (22.6)	26504 (77.4)	< 0.001
	< $50,000	29215 (26.1)	82807 (73.9)	
Population density	Q1 (most rural)	1011 (22.7)	3443 (77.3)	< 0.001
	Q2	2728 (25.4)	8019 (74.6)	
	Q3	4658 (26.1)	13179 (73.9)	
	Q4	5927 (26.2)	16701 (73.8)	
	Q5 (most urban)	18380 (24.8)	55647 (75.2)	
Physical activity in last 30 days	Yes	21343 (21.4)	78274 (78.6)	< 0.001
	No	15488 (33.6)	30647 (66.4)	
Green vegetable consumption	Below median (“Low”)	2032 (27.8)	5281 (72.2)	< 0.001
	Above median (“High”)	1460 (22.6)	4995 (77.4)	
Fruit consumption	Below median (“Low”)	1779 (28.5)	4466 (71.5)	< 0.001
	Above median (“High”)	1879 (23.2)	6232 (76.8)	
		*Mean (SD)*	*Mean (SD)*	
Age		72.6 (6.2)	75.2 (7.4)	< 0.001
Frequency of vegetable consumption per week		2.4 (1.9)	2.7 (2.1)	< 0.001
Frequency of fruit consumption per week		6.6 (5.7)	7.2 (7.0)	< 0.001
County per capita income ($ 000s)		27.3 (6.6)	28.0 (7.0)	< 0.001

Table 2 shows the percentage of respondents reporting having some physical activity in the prior month and their average weekly fruit and green vegetable consumption by population density quintile. With increasing urbanicity, the percentage of respondents participating in physical activity increased significantly (p < 0.001). There was also a significant positive association between weekly green vegetable consumption and rural-urban status (p = 0.021), but there was not an association between average weekly fruit consumption and population density (p = 0.775).

**Table 2:** Frequencies and descriptive statistics of physical activity and fruit and vegetable consumption by population density quintile.

		Percent physically active in last 30 days	Weekly vegetable consumption occasions	Weekly fruit consumption occasions
	Overall			
Population density quintile	Q1 (most rural)	66.4	2.8 (2.1)	6.4 (5.4)
	Q2	68.2	2.7 (1.9)	9.5 (31.8)
	Q3	66.0	2.5 (2.1)	5.3 (4.6)
	Q4	67.4	2.6 (2.0)	5.8 (5.1)
	Q5 (most urban)	69.7	2.8 (2.1)	6.0 (4.8)
		< 0.001	0.021	0.775

Odds ratios for the unadjusted and adjusted models of each of the four primary outcomes are shown in Table 3. Respondents in the most rural quintile (Q1) were significantly more likely to have obesity (OR 1.13, 95% CI 1.05, 1.21) than respondents in the most urban quintile (Q5). Obesity rates were significantly lower in Q3 and Q4 of population density (more rural) than in the most urban quintile (OR for Q3: 0.94, 95% CI 0.90, 0.97) (OR for Q4: 0.93, 95% CI 0.90, 0.96). After adjusting for confounders, obesity was significantly higher in the three most rural quintiles (Q1, Q2, Q3) than the most urban quintile (Q5). Low fruit consumption was more prevalent in the two most rural quintiles (Q1 & Q2) than in the most urban quintile in the adjusted models, and decreased with increasing urbanicity (p < 0.001).

**Table 3:** Model estimates of obesity, physical activity, and fruit and vegetable consumption based on population density quintile.

		Obesity	Physical activity	Low green vegetable consumption	Low fruit consumption
Unadjusted	Population density				
	Q1 (most rural)	1.13 (1.05, 1.21)	1.17 (1.10, 1.24)	1.01 (0.77, 1.32)	1.02 (0.87, 1.20)
	Q2	0.97 (0.93, 1.02)	1.08 (1.03, 1.12)	1.02 (0.76, 1.38)	1.13 (0.94, 1.35)
	Q3	0.94 (0.90, 0.97)	1.19 (1.15, 1.23)	0.74 (0.60, 0.93)	0.92 (0.81, 1.04)
	Q4	0.93 (0.90, 0.96)	1.12 (1.08, 1.15)	0.82 (0.71, 0.94)	0.93 (0.85, 1.01)
	Q5 (most urban, ref)	1	1	1	1
	P-value for trend	0.415	< 0.001	0.143	0.934
Adjusted	Population density				
	Q1	1.23 (1.14, 1.32)	1.05 (0.99, 1.13)	1.28 (0.96, 1.71)	1.25 (1.05, 1.48)
	Q2	1.06 (1.01, 1.12)	0.93 (0.89, 0.97)	1.27 (0.93, 1.73)	1.30 (1.08, 1.56)
	Q3	1.05 (1.01, 1.09)	1.00 (0.96, 1.04)	1.01 (0.80, 1.28)	1.14 (0.99, 1.30)
	Q4	1.03 (0.99, 1.07)	0.99 (0.96, 1.03)	0.97 (0.83, 1.13)	1.03 (0.94, 1.13)
	Q5 (ref)	1	1	1	1
	P-value for trend	< 0.001	0.342	0.074	< 0.001

The results of the second research objective—to assess whether the associations between predictors of obesity and obesity vary by rural-urban status—are shown in Table 4. Both unadjusted and adjusted models indicated a strong and significant association between physical activity and obesity regardless of rural-urban status. For the overall sample, obesity status was associated with low consumption of green vegetables (OR 1.33, 95% CI 1.17, 1.50) and low fruit intake (OR 1.22, 95% CI 1.13, 1.32). However, when stratified by population density quintile, the association between obesity status and low green vegetable consumption was significant only for the most urban quintile (Q5, OR 1.43, 95% CI 1.23, 1.67). Similar results were obtained for the association between low fruit consumption and obesity status, with the only significant association being in the most urban quintile (OR 1.27, 95% CI 1.15, 1.39). These results suggest that in older adults, lower green vegetable and fruit consumption is associated with increased obesity, but only in urban counties.

**Table 4:** Unadjusted and adjusted* odds ratios of obesity from physical activity, low vegetable consumption, and low fruit consumption overall and stratified by population density quintile.

		Overall	Quintile 1	Quintile 2	Quintile 3	Quintile 4	Quintile 5
Physical activity	Unadjusted	0.54 (0.53, 0.55)	0.59 (0.51, 0.68)	0.56 (0.51, 0.61)	0.55 (0.52, 0.59)	0.51 (0.48, 0.54)	0.52 (0.50, 0.54)
	Adjusted	0.50 (0.48, 0.52)	0.51 (0.44, 0.59)	0.52 (0.47, 0.57)	0.52 (0.49, 0.56)	0.48 (0.45, 0.51)	0.49 (0.47, 0.51)
Low green vegetable consumption**	Unadjusted	1.35 (1.20, 1.53)	1.03 (0.55, 1.92)	1.16 (0.60, 2.27)	1.38 (0.85, 2.22)	1.05 (0.79, 1.39)	1.47 (1.27, 1.71)
	Adjusted	1.33 (1.17, 1.50)	0.99 (0.52, 1.91)	1.33 (0.64, 2.77)	1.42 (0.86, 2.34)	1.08 (0.81, 1.44)	1.43 (1.23, 1.67)
Low fruit consumption**	Unadjusted	1.32 (1.23, 1.42)	1.08 (0.75, 1.55)	1.14 (0.75, 1.73)	1.30 (0.99, 1.69)	1.27 (1.07, 1.51)	1.37 (1.24, 1.50)
	Adjusted	1.22 (1.13, 1.32)	0.98 (0.67, 1.43)	1.05 (0.67, 1.63)	1.23 (0.94, 1.62)	1.15 (0.96, 1.37)	1.27 (1.15, 1.39)

*Adjusted for gender, race/ethnicity, education level, age, personal income, and county-level per capita income

**Based on median values: Below 0.5 servings per day of green vegetables and below 1 serving per day of fruit

## Discussion

Study findings suggest that there are important associations between rural-urban status and obesity and obesity-related factors for older adults. Results of this current study largely support results of previous research that show that for adults of all ages, obesity rates are higher in rural areas than in urban areas [35–39], while low fruit and vegetable consumption and rates of physical activity are lower in rural areas compared to urban areas [45]. There are several potential explanations for these findings. For physical activity, rural older adults may be less likely to participate in physical activity than their urban counterparts because there may be fewer designated places for physical activity, such as neighborhood streets with sidewalks, senior centers, walking paths, parks, and malls. Rural older adults are also more likely to report being in poor health than urban or suburban older adults, which may make them less likely to be physically active. Furthermore, due to physical distance, they also may have lower social support for exercise and physical activity than their urban counterparts [46]. Actual availability of grocery stores in close proximity and lack of access to transportation to grocery stores [47] may limit the availability of fruits and vegetables and other healthful foods for older adults in rural areas as they may depend on smaller convenience stores than residents in cities [48]. Also, the rural poor have fewer choices in food outlets than their urban counterparts [49], creating food deserts. Rural older adults would have those same limited options in availability of transportation, food outlets, and grocery stores.

The results of this study support the vast body of research showing that low levels of physical activity, low fruit consumption and low green vegetable consumption are associated with a higher likelihood of obesity. However, the results of the current study adds the existing body of knowledge on the established associations between obesity and both diet and physical activity and indicate that these known associations vary by rural-urban status. Although the association between physical activity and obesity remained consistent and significant, regardless of rural-urban status, the well-established associations between obesity and fruit and green vegetable consumption were strongest in urban areas, and not significant in more rural areas. The reasons for these findings are not clear. Dietary patterns are complex and extend beyond basic measures of fruit and green vegetable consumption used on this study. There may be other factors, dietary or other, that promote obesity in rural older adults distinct from low fruit and green vegetable consumption that may differ between rural and urban older adults, including other socioeconomic and demographic factors, such as income and education, which themselves vary by rural-urban status [50]. Furthermore, there may be rural-urban differences in terms of physical activity performed as part of one’s occupation. It is possible that people living in the most rural areas may be more likely to be involved with agriculture and related occupations and activities, which themselves provide opportunities for physical activity. Future research should explore these potential relationships.

Study findings should be interpreted with several important limitations and caveats in mind. First, this is a secondary analysis of cross-sectional data and causality cannot be determined. Second, as this is a secondary data analysis, questions on dietary patterns, physical activity, and obesity were limited to the information available in the dataset. Only a limited number of factors potentially related to obesity (physical activity and consumption of fruit and green vegetables) were available in the data set and examined in this analysis. Third, all data in this study were ascertained through self-report and may be subject to several types of bias, including social desirability bias [51]. Relatedly, the cutoff value for BMI of 30 kg/m^2^ was used to categorize respondents by obesity in this study. In studies of older adults, obesity is classified as having a BMI of over 32 kg/m^2^. Had the latter cutoff been used, the results could have changed to some extent [52]. Another limitation is the rural-urban status measurement: Population density was used as the measure of rural-urban status. However, what defines “rural” and “urban” is more complex and multifaceted than simply population density [53–55]. Also, rural-urban status was assessed on the county level, but may have different impacts on other geographic levels, such as the block group, census tract, or state [56]. Furthermore, assessment of sociodemographic factors on the county level presents challenges in health research; U.S. counties are largely administrative—i.e. they are not specifically designed for research purposes—and tend to be heterogeneous in terms of size, structure, function, and layout [57]. The associations observed on the county level may not coincide with the associations that may exist at other geographic levels. Additionally, no geospatial modeling was conducted in this study due to gaps in the geographic coverage of older adults in the BRFSS across the country. Lastly, although age was considered in this study as a confounder, there is suggestive evidence that the effects of obesity on health and mortality may actually reverse as people age and that in the oldest segments of the population, particularly at and above age 80, obesity may actually be protective against mortality [58]. Future research could involve stratifying the sample by age group to explore potential differences in obesity precursors and potential effects, such as mortality, by age.

Despite these limitations, there are several notable strengths of this study. First, this is the first national study to address the potential for the well-established associations between physical activity and dietary habits and obesity to vary based on rural-urban status. Second, this study used a large, nationally representative sample of adults aged 65 and above to both substantiate previous findings of associations between rural-urban status and obesity and related predictors of obesity, as well as examine the potential for associations between obesity and obesity-related behaviors to vary by rural-urban status. Specifically, this analysis considered both monotonic and non-monotonic associations for both objectives.

## Conclusions

Obesity has important implications for the health of older adults, and is an important public health issue in the U.S. [16]. Its importance will likely only increase in the coming decades due to the aging of the “baby boomer” cohort, those born between 1946 and 1946, who are, by many measures, less healthy as a population than preceding cohorts. Baby boomers were 32% more likely to obese than members of the previous generation, and were three times more likely to not engage in any physical activity than the previous generation [59]. The increase in obesity prevalence from previous generations observed among baby boomers has likely undermined improvements in health that might have otherwise occurred during their lifetime [60]. Thus, there is a critical public health need to identify and address the causes of overweight and obesity among baby boomers as that cohort ages to prevent or perhaps reverse the trends in increasing obesity that otherwise promise to increase demand upon an increasingly strained the health care system in the coming decades [61].

The main findings of this study provide further evidence that interventions, policies, and programs designed at addressing the social, economic, and environmental causes of obesity in older adults should be tailored to address the unique needs of rural and urban older adults as interventions, policies, and programs that may be effective in urban areas may be less effective in rural areas or vice-versa. Specifically, study results suggest that efforts to promote eating fruits and vegetables and perhaps other healthy foods may not have as high as an impact in rural areas compared to urban areas. Future research should focus on why these findings occur and what can be done about them to improve interventions and policies for health promotion and obesity prevention among older adults. Growing evidence suggests that rural older adults face unique challenges with respect to health, health-seeking behaviors, and health care access and quality. One-size-fits all approaches to solving the obesity epidemic in the current or future generations of older adults will likely not achieve maximum effectiveness. To maximize effectiveness and reduce rural-urban disparities in vulnerable older adults, all such interventions and policies should be tailored to meet the unique barriers and needs specific to rural and urban older adults they are intending to support.
